# Novel Functional MRI Task for Studying the Neural Correlates of Upper Limb Tremor

**DOI:** 10.3389/fneur.2018.00513

**Published:** 2018-07-02

**Authors:** Frederique M. C. Boonstra, Thushara Perera, Gustavo Noffs, Cassandra Marotta, Adam P. Vogel, Andrew H. Evans, Helmut Butzkueven, Bradford A. Moffat, Anneke van der Walt, Scott C. Kolbe

**Affiliations:** ^1^Department of Medicine, University of Melbourne, Melbourne, VIC, Australia; ^2^The Bionics Institute, East Melbourne, VIC, Australia; ^3^Department of Medical Bionics, University of Melbourne, Melbourne, VIC, Australia; ^4^Department of Neurology, Royal Melbourne Hospital, Melbourne, VIC, Australia; ^5^Centre for Neuroscience of Speech, University of Melbourne, Melbourne, VIC, Australia; ^6^Redenlab, Melbourne, VIC, Australia; ^7^Department of Neurodegeneration, Hertie Institute for Clinical Brain Research, University of Tübingen, Tübingen, Germany; ^8^Department of Medicine, Melbourne Brain Centre, University of Melbourne, Melbourne, VIC, Australia; ^9^Department of Neuroscience, Central Clinical School, Monash University, Clayton, VIC, Australia; ^10^Florey Institute of Neuroscience and Mental Health, Parkville, VIC, Australia

**Keywords:** tremor, upper limb, fMRI, validation, reproducibility

## Abstract

**Introduction:** Tremor of the upper limbs is a disabling symptom that is present during several neurological disorders and is currently without treatment. Functional MRI (fMRI) is an essential tool to investigate the pathophysiology of tremor and aid the development of treatment options. However, no adequately or standardized protocols for fMRI exists at present. Here we present a novel, online available fMRI task that could be used to assess the *in vivo* pathology of tremor.

**Objective:** This study aims to validate the tremor-evoking potential of the fMRI task in a small group of tremor patients outside the scanner and assess the reproducibility of the fMRI task related activation in healthy controls.

**Methods:** Twelve HCs were scanned at two time points (baseline and after 6-weeks). There were two runs of multi-band fMRI and the tasks included a “brick-breaker” joystick game. The game consisted of three conditions designed to control for most of the activation related to performing the task by contrasting the conditions: WATCH (look at the game without moving joystick), MOVE (rhythmic left/right movement of joystick without game), and PLAY (playing the game). Task fMRI was analyzed using FSL FEAT to determine clusters of activation during the different conditions. Maximum activation within the clusters was used to assess the ability to control for task related activation and reproducibility. Four tremor patients have been included to test ecological and construct validity of the joystick task by assessing tremor frequencies captured by the joystick.

**Results:** In HCs the game activated areas corresponding to motor, attention and visual areas. Most areas of activation by our game showed moderate to good reproducibility (intraclass correlation coefficient (ICC) 0.531–0.906) with only inferior parietal lobe activation showing poor reproducibility (ICC 0.446). Furthermore, the joystick captured significantly more tremulous movement in tremor patients compared to HCs (p = 0.01) during PLAY, but not during MOVE.

**Conclusion:** Validation of our novel task confirmed tremor-evoking potential and reproducibility analyses yielded acceptable results to continue further investigations into the pathophysiology of tremor. The use of this technique in studies with tremor patient will no doubt provide significant insights into the treatment options.

## Introduction

Effective and efficient use of hands and arms is essential for undertaking daily activities (e.g., writing or consuming food and drink). Tremulous movements of the upper limb while performing activities of daily living, specifically action tremors, are a symptom of a variety of neurological disorders, including Essential Tremor (ET) and multiple sclerosis (MS). These movements vary in severity but even when mild, can interfere with daily activities and be perceived by the patient as severely embarrassing (1, 2). Ameliorating tremor is therefore a priority for patients and clinicians treating these diverse diseases. While several treatments are in routine use, medical therapy often fails to provide adequate control (3). The pathogenesis of tremors remains incompletely understood, further limiting the development of treatment options.

Action tremor encompasses tremors involve in movement, posture against gravity, and movement toward a target (intention tremor). These types of tremor arises from problems in parts of the brain that control and integrate movement. In ET and MS, most studies into the pathophysiology of tremor use surgical, post-mortem, neurophysiological, or animal studies. The use of magnetic resonance imaging (MRI) has been limited to structural brain imaging or basal network activity using resting-state functional MRI (fMRI). These studies have implicated the cerebellum, thalamus, red nucleus and cortex (4–9). Few studies have investigated the pathogenesis and relevant functional neuroanatomy of tremulous movement using fMRI.

Assessing *in vivo* brain activation via fMRI, especially during a movement/task, provides for a unique opportunity to better understand the pathophysiology of neurological upper limb function. However, the utility of fMRI in this field is under-explored. The most commonly used fMRI tasks to examine action tremor are the finger-tapping movement, finger-flexion and hand gripping task (10–16). These simple tasks may not capture the full extent of the tremor that usually involve oscillations around several joints, including the shoulder joint, elbow, wrist and fingers, and the tasks are often not complex enough to represent typical, everyday manual movements during which the upper limb dysfunction is most noticeable. Furthermore, tasks are often not standardized, with variable study designs in the absence of proper control tasks to separate the activity related to performing the task from activity related to the upper limb dysfunction within the patients (17). Reproducibility is essential for the implementation of any proposed task in longitudinal studies, particularly when attempting to study the effect of treatment. It is important to study reproducibility to determine measurement error and sensitivity prior to application in patient groups, however, limited data is available about variability and reproducibility of tasks used to examine action tremor (18, 19).

We developed a novel, online available task that is dynamic enough to elicit a tremor independently of the upper limb area where the tremor is evident. In this study, we aimed to assess the feasibility of this task, potentially useful for studying the neural correlates of action tremor in the MRI scanner. To this end, we aimed to show proof of validity that our task evokes tremor in patients with action tremor. In addition, we examine the brain networks activated by a novel fMRI task and test the reproducibility of these activations in a group of healthy individuals. Developing a task that is able to reproducibly activate brain regions associated with upper limb tremor is an essential first step in exploring the brain regions involved in tremor and testing putative neural effects of treatments that ameliorate tremor.

## Methods

### Participants

Twelve healthy control (HC) participants (45.4 ± 15.3 y, 75% female) underwent testing at baseline and 6-week follow-up that included performance of a joystick task both outside and inside the MRI scanner. In order to validate that the joystick task was able to evoke tremulous motion, we recruited four patients with multiple sclerosis and unilateral right-sided upper limb tremor [49.3 ± 4.3 y, 75% female, median Expanded Disability Status Scale (EDSS) 5.25, interquartile range (IQR) 3.75 (20); and median Bain Tremor Rating 4.5, IQR 4 (21, 22)]. The patients performed the joystick task with the affected hand outside of the scanner. The study was approved by the local Human Research Ethics Committee and all participants provided voluntary written consent.

### MRI acquisition

**Figure 1:**
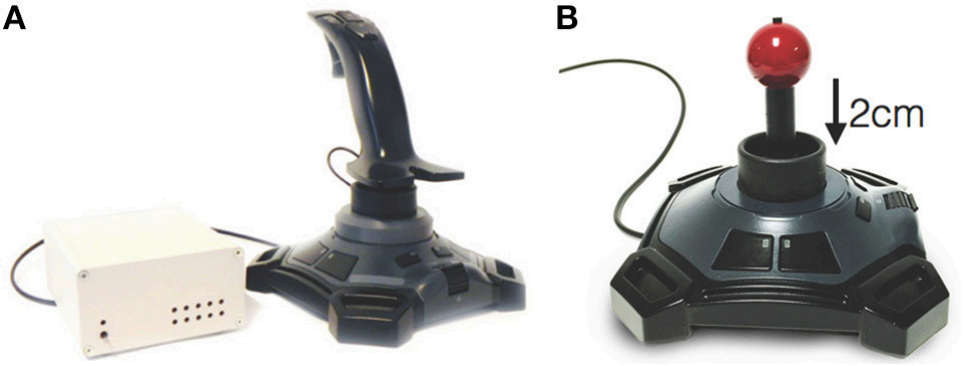
The joystick used outside of the scanner **(A)** and inside the scanner **(B)**. The right figure shows the stick that we have shortened to facilitate use inside the scanner.

Healthy controls underwent 3T MRI (MAGNETOM TrioTim, Siemens, Medical Systems, Erlangen, Germany) to acquire: (a) T1-weighted volumetric sequence (TR = 11.0 ms, TE = 5.0 ms, FOV = 1536^*^1536 mm^2^, matrix = 208^*^256, slice thickness = 8.0 mm, flip angle = 7.0°), and (b) two runs of gradient echo planar imaging fMRI (TR = 1.5 ms, TE = 33 ms, FOV = 204^*^204 mm^2^, matrix = 104^*^104, slice thickness = 2 mm, flip angle = 85.0°, volumes = 200, GRAPPA acceleration factor of 2, multi-band slice acceleration factor of 3). We used a MRI compatible joystick (FOJ-2B-10B, NAtA technologies) with 67 Hz sampling frequency of x and y joystick positions (Figure 1). To facilitate use of the joystick inside the MRI bore, the handle was shortened (Figure 1B). During imaging, the joystick was stabilized on the subject's torso using a wedge-shaped foam pillow that allowed the subject to lie in a more comfortable position.

### Joystick task

The joystick task was purpose-written in C# (Visual Studio 2010, Microsoft Corporation, Washington, USA) and incorporates a game adapted from “Brick Ball Game” (23). The game timing was controlled by the MRI triggers. A compiled version is available for download from GitHub^1^. The task is based on the retro arcade game *Breakout (Atari, Inc.)*. The objective is to manipulate a paddle along the bottom of the screen to bounce a ball onto bricks. Bricks break and disappear from the screen when the ball collides with them. We modified this game in three ways: (1) sufficient rows of bricks were added to the top of the screen such that the player would never be able to break all of them within a single trial, (2) gameplay continues even after a player has failed to maneuver the paddle to correctly bounce the ball, and (3) two balls (white and red) were introduced, with one being a distractor. Points 1 and 2 above allow the game to continue without pauses and remain consistent across trials.

The game was a block design with three conditions: PLAY (playing the game), WATCH (watching a simulated game without moving the joystick to control for visual attention related activity) and MOVE (moving the joystick left and right across the entire screen at a self-timed pace to control for basic manual motor activity). Each task block commenced with the display of an instruction screen indicating which condition was to follow for 5 s. Each condition was presented three times for 30 s, and the condition order was randomized.

This task should provide the complexity that allows a tremor to appear in patients with neurological tremor. Importantly, the participant's arm movement was not restricted during our study and we did not explicitly instruct participants to move the joystick using wrist alone. Tremor originating in the forearm and upper-arm will mechanically couple through the wrist joint and be measurable with the joystick (i.e., the hand is the end-effector and its position is influenced by any movement of connected joints: elbow and shoulder). The task was performed during fMRI scanning by HC subjects, and the start of the game was triggered by a pulse sent from the MRI scanner. The game was performed under the same conditions outside the scanner by all subjects using the full-sized joystick shown in Figure 1A.

### Joystick task analysis

During performance of the task, the position of on-screen objects, time timestamps for each MRI trigger and joystick movements were recorded. The joystick data were pass-band filtered (3–10 Hz) to attenuate non-tremulous activity using custom Matlab scripts. The average power of the remaining frequency band (expressed in decibels, dB) was used to represent the amplitude of tremor-like movement. To validate that the task was able to elicit tremulous movements in patients with a diagnosed neurological tremor, we measured the amount of tremor-like movement in four patients with MS. As the patients performed the joystick task outside of the scanner, we compared this with the amount of tremor-like movement in HC when they performed the task outside the scanner. Differences in tremor-like movement were calculated using one-way ANOVA. Finally, we assessed whether there was a change in tremor-like movement over time in HCs.

### Imaging analysis

Functional MRI analyses were performed using FEAT v6.00 (FSL, FMRIB, Oxford, UK). Raw fMRI scans were pre-processed to correct for head motion, spatially smoothed (4 mm extent threshold) and registered to the main structural image using boundary based registration and then to standard MNI space using FNIRT nonlinear registration.

Given that two conditions in the task involved hand movements that could induce head motion, we investigated the degree of head motion in all subjects. FSL motion outlier was used to detect slices within the data that was corrupted by large movements, with threshold set at the 75th percentile + 1.5 times the interquartile range. The confound matrix resulting from motion artifact detection was subsequently used in a general linear model (GLM) to remove the effects of these slices on the analysis.

Multi-level GLM analysis was used to identify regions of significant activation at baseline in HC subjects for the following contrasts:

1. PLAY > WATCH (motor component)
2. PLAY > MOVE (visual-attention component)
3. PLAY > WATCH + MOVE.

Run-level environment variables included the time-course for each condition and associated temporal dispersion derivatives, and head translation and rotation parameters. Relative movement was calculated as the relative difference in head position and rotation volume to volume. Second-level analyses were used to combine the two joystick runs performed for each subject. Finally, third-level analyses assessed common areas of brain activation across all HC subjects.

### Reproducibility

To assess reproducibility, we compared the peak activation within clusters identified during baseline with data from the 6-week follow-up. From the third-level GLM analyses, we extracted the significant group level clusters for all three contrasts at baseline. These clusters were used to identify the maximum z-stat within each cluster for each HC at both baseline and at the 6-week scans. Reproducibility was quantified using two separate measures: intraclass correlation [ICC; based on a mean of measures, absolute agreement, 2-way mixed-effect model (24)] for a measure of repeatability and the average coefficient of variation (CoV) to quantify the amount of variation useful for future treatment studies. For the ICC, we compared the maximum z-stat for all the clusters for each HC between baseline and the 6-week time point. For the CoV we calculated the temporal standard deviation for the baseline and 6-week time points for each HC and divided this by the mean of both time points. The final CoV per cluster was the average of the CoV for all HCs.

## Results

### Head movement

The relative motion and the number of slices removed for each subject are shown in Supplementary Table 1. At baseline, the median of relative movement was 0.105 mm and the maximum numbers of slices removed was 7.5% (30 out of 400 slices). At 6 weeks, the average median of relative movement was 0.102 mm and the maximum number of slices removed was 7% (28 out of 400 slices).

### Validation of tremulous motion during task performance in tremor patients

**Figure 2:**
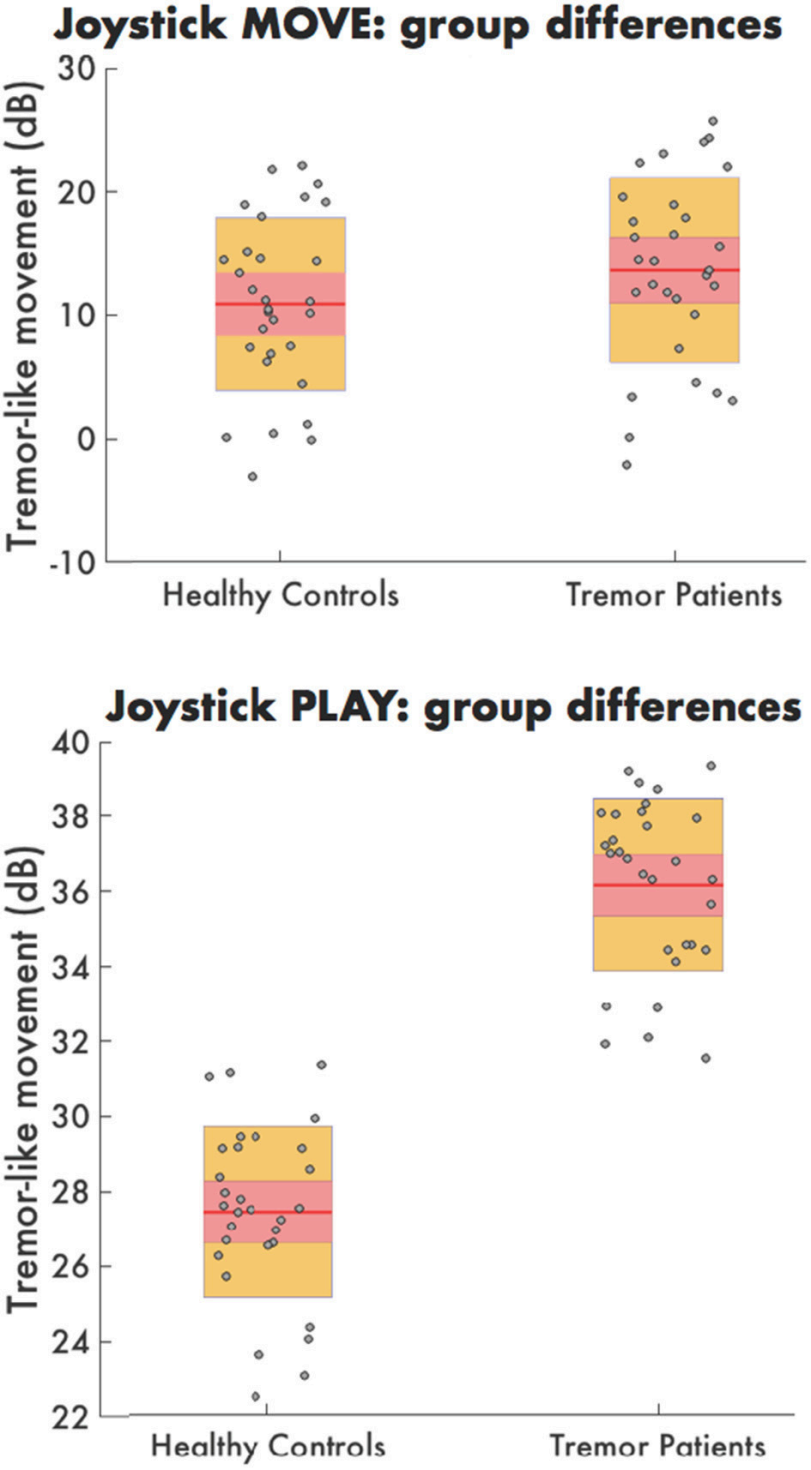
The tremor-like movement during MOVE (**top**) PLAY (**bottom**) for both healthy controls and tremor patients. Each dot represents the average tremor-like movement per time point for the healthy controls (**left**) and tremor patients (**right**) during the PLAY condition.

Average power in the tremor-like frequency band for joystick motion during MOVE was not significantly different in HC (10.8 dB) compared to tremor patients (14.9 dB) (*F* = 0.08, *p* = 0.787). However, during PLAY, the amount of tremor-like movement was significantly higher in tremor affected MS patients (36.2 dB) than for HC (27.9 dB) (*F* = 9.53, *p* = 0.010) (Figure 2).

Finally, the amount of tremor-like movement was consistent over time in HC with no significant change at baseline vs. 6-week follow-up during PLAY (*t* = 1.528, *p* = 0.155) and MOVE (*t* = 0.598 *p* = 0.5618).

### Task analyses

**Figure 3:**
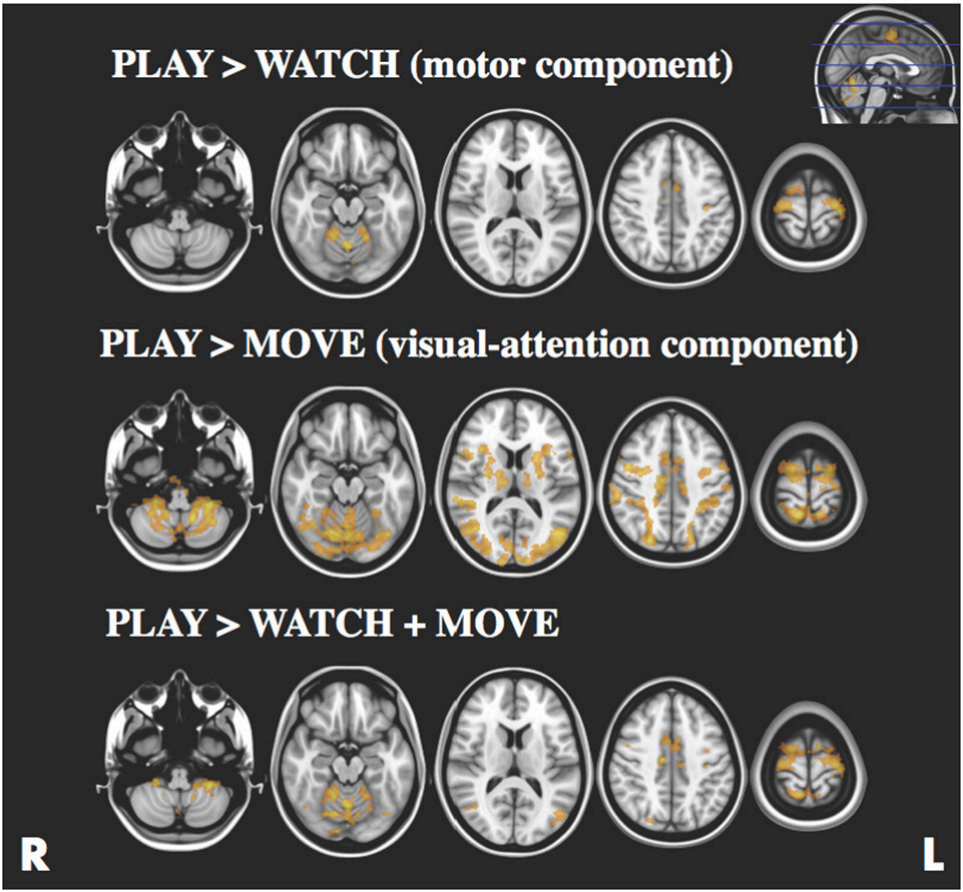
Statistical parametric maps for the PLAY > WATCH (**top**), PLAY > MOVE (**middle**) and PLAY > WATCH + MOVE (**bottom**) contrasts, z-stat threshold of >2.3. Images are presented according to radiological convention (R = right, L = left).

The statistical maps for PLAY > WATCH, PLAY > MOVE and PLAY > WATCH + MOVE are presented in Figure 3 and Table 1. At baseline, we found four significant (z-stat > 2.3) clusters during PLAY + WATCH. Specifically, activation cluster 1.1 and 1.2 were part of the contralateral and ipsilateral motor cortex respectively. Activation cluster 1.3 and 1.4 were part of the ipsilateral and contralateral cerebellum respectively.

**Table 1:** Significant clusters and their corresponding activation peaks for the PLAY > WATCH, PLAY > MOVE and PLAY > WATCH +MOVE contrasts x, y, z, the average MNI coordinates of the maximum activation peak for all HC for each clusters; ICC, intraclass correlation; CoV, coefficient of variation; R, right hemisphere; L, left hemisphere.

**Cluster #**	**Voxels**	**Anatomical region**		**MNI coordinates (mm)**			**ICC**	**CoV (%)**
				**x**	**y**	**z**		
**PLAY** > **WATCH** + **MOVE**								
1	10,964	Motor cortex Superior parietal lobe	L/R	−11	−18	63	0.850	12.9
2	5,740	Cerebellum	L/R	−22	−65	−9	0.888	11.3
3	673	Visual cortex	R	47	−67	4	0.689	25.6
4	376	Inferior parietal lobe	R	63	−30	28	0.446	19.5
**PLAY** > **WATCH**								
1	2,781	Motor cortex	L	−24	−14	78	0.823	19.2
2	2,393	Motor cortex	R	46	−2	74	0.624	18.3
3	968	Cerebellum	R	28	−42	−10	0.881	21.0
4	478	Cerebellum	L	−14	−40	−14	0.531	29.3
**PLAY** > **MOVE**								
1	37,689	Visual–attention network	L	50	8	70	0.906	8.6
2	626	Frontal operculum cortex	R	54	32	24	0.692	23.4
3	498	Frontal operculum cortex	L	−20	22	20	0.536	23.0
4	317	Motor cortex	L	−44	12	42	0.743	20.6

During PLAY > MOVE we found four significant (z-score > 2.3) clusters. Specifically, the first activation cluster 2.1 was part of the visual attention network. Activation cluster 2.2 and 2.3 were part of the ipsilateral and contralateral frontal operculum cortex. Finally, activation cluster 2.4 was within the contralateral motor cortex.

Finally, we found four significant (z-score > 2.3) clusters during the PLAY > WATCH + MOVE condition. Specifically, the first activation cluster 3.1 was part of the contralateral and ipsilateral motor cortex that includes the contralateral precentral and postcentral gyrus, and it extended toward the superior parietal lobe. The second activation cluster 3.2 includes ipsilateral and contralateral cerebellum. The third activation cluster 3.3 was in the ipsilateral visual cortex. The fourth activation cluster 3.4 was within the inferior parietal lobe.

### Reproducibility

Reproducibility measures, ICC and CoV, between baseline and 6-week follow-up was calculated for all clusters and are shown in Table 1. The ICC ranged from 0.437 to 0.888 and the CoV ranged from 9.2 to 28.2%.

## Discussion

This study aimed to validate a novel fMRI task potentially useful to study the brain activation correlates of upper limb tremor. Using a test condition (PLAY) and two control conditions (MOVE and WATCH) we were able to differentially identify regions involved in motor and visual attention aspects of the task. Motor regions included pre- and post- central gyrus and the cerebellum. These regions of activation are similar to previous upper limb motor studies in healthy controls that indicate involvement of the premotor cortex, supplementary motor cortex, primary motor cortex and the cerebellum (25, 26). Visual regions included occipital cortex, occipital pole and lingual gyrus. These regions are known to play a role in visual processing (27). Evidence suggests that tremors can originate after an insult or injury to the cerebello-thalamo-cortical tract (13, 28, 29). Our task showed activation within areas of both ipsilateral and contralateral cerebellum that are known to be functionally linked to the somatomotor and premotor cortex (30). However, no thalamic activations were detected indicating insignificant role of the thalamus during this task. Taken together, we believe that our work demonstrates an upper limb motor task that successfully activates the motor network, including areas hypothesized to be involved in action tremor pathophysiology.

We have shown that all brain areas activated by our task have moderate to good reliability, with the exception of the inferior parietal lobe (31). Specifically, we found good reproducibility of the fMRI activation in the contralateral motor cortex and ipsilateral cerebellum, which indicates a strong activation within these brain areas. Our ICC within the motor cortex is consistent with ICC found by non-tremor focused upper-limb imaging studies (32–34). Furthermore, other studies that have used the CoV to assess the reproducibility of BOLD signals have also shown similar results (35, 36); specifically, (36) examined the CoV for the primary motor cortex and found a group mean of 24%. Reproducibility is essential to facility longitudinal use of the joystick task in future studies.

Importantly, patients with diagnosed upper-limb tremor displayed a significant increase in tremulous movements during the task compared to HCs, validating the ability of the task to elicit tremulous movements in future clinical applications. Tremor is one of the most common movement disorders and is a symptom of many neurological disorders including PD, ET, and MS (37, 38). In MS, tremor often presents as an action tremor with a postural and intention tremor component (39). There have been no task fMRI studies in MS tremor patients and tasks used in fMRI studies of other neurological diseases causing upper limb dysfunction lack the complexity to elicit tremulous movement and ability to assess tremor in the whole upper limb. For example, finger-tapping has been used in PD tremor and ET but a tremor is not produced during the task, possibly due to the low muscle activation (11). MS patients with tremor experienced more tremor-like movement while playing the game compared to controls, indicating our task successfully evokes tremulous movement. Examining the brain activity while patients are experiencing tremulous movement would be the most informative about the pathophysiology of intention tremors in neurological disorders, including MS patients.

Understanding the pathophysiology of PD and ET have led to improved and targeted treatments for these disorders such as deep brain stimulation (DBS) of the sub-thalamic area. DBS in this specific area disrupts pathological circuits in the cerebello-thalamo-cortical and pallido-thalamo-cortical pathways (40). The knowledge of this motor circuitry is currently based on animal and surgical studies (40, 41–44). The value of fMRI is the non-invasive method to image motor circuitry and networks *in vivo*. To maximize its use, experiments need to be designed meticulously and validation and reproducibility assessments are essential.

Two limitations of our study are the low number of subjects and having only two different time points to assess reproducibility. Increasing the sample size of our study could provide more power to assess the reproducibility of the joystick task. By providing more time points we could better assess a learning effect over time. Further studies in larger cohorts and subsequent time points will allow a more thorough investigation of the reproducibility and ability to control for task related activation.

To conclude, our study introduced a novel task for fMRI studies that focuses on neurological upper limb dysfunction. Reproducibility shows acceptable results, and importantly our task is properly controlled and showed ecological validity. Future studies using this joystick task in patients with upper-limb tremors will further elucidate the pathophysiology of tremor.

## Author contributions

FB, TP, and SK has worked on study design, data collection, data analyses, data interpretation and writing the manuscript. GN and CM has worked on data collection and writing the manuscript. AV, AE, BM, and HB has worked on writing the manuscript. AvdW has worked on study design, data collection, data interpretation and writing the manuscript.

### Conflict of interest statement

The authors declare that the research was conducted in the absence of any commercial or financial relationships that could be construed as a potential conflict of interest.

## Footnotes

^1^https://github.com/FBoonstra/Joystick-fMRI

## References

[1] Lorenz D Schwieger D Moises H Deuschl G. Quality of life and personality in essential tremor patients. Mov Disord (2006) 21:1114–8. 10.1002/mds.20884 16622851

[2] Louis ED Rios E. Embarrassment in essential tremor: prevalence, clinical correlates and therapeutic implications. Parkinsonism Relat Disord. (2009) 15:535–8. 10.1016/j.parkreldis.2008.10.006 19028131 PMC2712578

[3] Schneider SA Deuschl G. Medical and surgical treatment of tremors. Neurol Clin. (2015) 33:57–75. 10.1016/j.ncl.2014.09.005 25432723

[4] Benninger DH Thees S Kollias SS Bassetti CL Waldvogel D. Morphological differences in Parkinson's disease with and without rest tremor. J Neurol. (2009) 256, 256–63. 10.1007/s00415-009-0092-2 19219572

[5] Boonstra F Florescu G Evans A Steward C Mitchell P Desmond P. Tremor in multiple sclerosis is associated with cerebello-thalamic pathology. J Neural Transm. (2017) 124:1509–14. 10.1007/s00702-017-1798-4 29098451 PMC5686246

[6] Helmich RC Janssen MJR Oyen WJG Bloem BR Toni I. Pallidal dysfunction drives a cerebellothalamic circuit into Parkinson tremor. Ann Neurol. (2011) 69:269–81. 10.1002/ana.22361 21387372

[7] Jia L Jia-Lin S Qin D Qing L Yan Z. A diffusion tensor imaging study in essential tremor. J Neuroimaging (2011) 21:370–4. 10.1111/j.1552-6569.2010.00535.x 21091815

[8] Klein JC Lorenz B Kang JS Baudrexel S Seifried C van de Loo S. Diffusion tensor imaging of white matter involvement in essential tremor. Hum Brain Mapp. (2011) 32, 896–904. 10.1002/hbm.21077 20572209 PMC6870356

[9] Saini J Bagepally BS Bhatt MD Chandran V Bharath RD Prasad C. Diffusion tensor imaging: tract based spatial statistics study in essential tremor. Parkinsonism Relat Disord. (2012) 18:477–82. 10.1016/j.parkreldis.2012.01.006 22297126

[10] Bucher SF Seelos KC Dodel RC Reiser M Oertel WH. Activation mapping in essential tremor with functional magnetic resonance imaging. Ann Neurol. (1997) 41:32–40. 10.1002/ana.410410108 9005863

[11] Buijink AW Broersma M van der Stouwe AM van Wingen GA Groot PF Speelman JD. Rhythmic finger tapping reveals cerebellar dysfunction in essential tremor. Parkinsonism Relat Disord. (2015) 21, 383–8. 10.1016/j.parkreldis.2015.02.003 25703340

[12] Buma FE Raemaekers M Kwakkel G Ramsey NF. Brain function and upper limb outcome in stroke: a cross-sectional fMRI study. PLoS ONE 10:e0139746. 10.1371/journal.pone.0139746 26440276 PMC4595281

[13] Lewis MM Du G Sen S Kawaguchi A Truong Y Lee S. Differential involvement of striato- and cerebello-thalamo-cortical pathways in tremor-and akinetic/rigid-predominant Parkinson's disease. Neuroscience (2011) 177, 230–9. 10.1016/j.neuroscience.2010.12.060 21211551 PMC3049982

[14] Lewis MM Slagle CG Smith AB Truong Y Bai P McKeown MJ. Task specific influences of Parkinson's disease on the striato-thalamo-cortical and cerebello-thalamo-cortical motor circuitries. Neuroscience (2007) 147, 224–35. 10.1016/j.neuroscience.2007.04.006 17499933 PMC1939944

[15] Manganotti P Acler M Formaggio E Avesani M Milanese F Baraldo A. Changes in cerebral activity after decreased upper-limb hypertonus: an EMG-fMRI study. Magn Reson Imaging (2010) 28, 646–52. 10.1016/j.mri.2009.12.023 20117894

[16] Tessa C Diciotti S Lucetti C Baldacci F Cecchi P Giannelli M. fMRI changes in cortical activation during task performance with the unaffected hand partially reverse after ropinirole treatment in de novo Parkinson's disease. Parkinsonism Relat Disord. (2013) 19, 265–8. 10.1016/j.parkreldis.2012.07.018 22901957

[17] Sharifi S Nederveen AJ Booij J van Rootselaar AF. Neuroimaging essentials in essential tremor: a systematic review. NeuroImage Clin. (2014) 5:217–31. 10.1016/j.nicl.2014.05.003 25068111 PMC4110352

[18] McKinsey RD Moritz CH Meyerand ME Tome WA. Assessment of multiple task activation and reproducibility in patients with benign and low-grade neoplasm. Technol Cancer Res Treat. (2010) 9:319–26. 10.1177/153303461000900402 20626198 PMC2906819

[19] Yoo SS O'Leary HM Lee JH Chen NK Panych LP Jolesz FA. Reproducibility of trial-based functional MRI on motor imagery. Int J Neurosci. (2007) 117:215–27. 10.1080/00207450600582546 17365109

[20] Kurtzke JF. Rating neurologic impairment in multiple sclerosis: an expanded disability status scale (EDSS). Neurology (1983) 33, 1444–1452. 6685237 10.1212/wnl.33.11.1444

[21] Alusi S Worthington J Glickman S Findley L Bain P. Evaluation of three different ways of assessing tremor in multiple sclerosis. J Neurol Neurosurg Psychiatry (2000) 68:756–60. 10.1136/jnnp.68.6.756 10811700 PMC1736951

[22] Bain PG Findley LJ Atchison P Behari M Vidailhet M Gresty M. Assessing tremor severity. J Neurol Neurosurg Psychiatry (1993) 56, 868–873. 8350102 10.1136/jnnp.56.8.868 PMC1015140

[23] Gajjar A. Brick Ball Game: WPF# Game. (2014). Available online at: https://www.codeproject.com/Articles/470039/Brick-Ball-Game-WPF-Csharp-Game

[24] Koo TK Li MY. A guideline of selecting and reporting intraclass correlation coefficients for reliability research. J Chiropract Med. (2016) 15:155–63. 10.1016/j.jcm.2016.02.012 27330520 PMC4913118

[25] Cowper-Smith CD Lau EYY Helmick CA Eskes GA Westwood DA. Neural coding of movement direction in the healthy human brain. PLoS ONE 5:e13330. 10.1371/journal.pone.0013330 20967197 PMC2954155

[26] Dhamala M Pagnoni G Wiesenfeld K Zink CF Martin M Berns GS. Neural correlates of the complexity of rhythmic finger tapping. NeuroImage (2003) 20:918–26. 10.1016/S1053-8119(03)00304-5 14568462

[27] Bisley JW The neural basis of visual attention. J Physiol. (2011) 589(Pt 1), 49–57. 10.1113/jphysiol.2010.192666 20807786 PMC3039259

[28] Luo C Song W Chen Q Yang J Gong Q Shang HF. White matter microstructure damage in tremor-dominant Parkinson's disease patients. Neuroradiology (2017) 59:691–8. 10.1007/s00234-017-1846-7 28540401

[29] Sen S Kawaguchi A Truong Y Lewis MM Huang X. Dynamic changes in cerebello-thalamo-cortical motor circuitry during progression of Parkinson's disease. Neuroscience (2010) 166:712–9. 10.1016/j.neuroscience.2009.12.036 20034546 PMC2852615

[30] Buckner RL Krienen FM Castellanos A Diaz JC Yeo BTT. The organization of the human cerebellum estimated by intrinsic functional connectivity. J Neurophys (2011) 106:2322–45. 10.1152/jn.00339.2011 21795627 PMC3214121

[31] Portney LG Watkins MP Foundations of Clinical Research: Application to Practice. Prentice Hall (2000).

[32] Caceres A Hall DL Zelaya FO Williams SC Mehta MA. Measuring fMRI reliability with the intra-class correlation coefficient. NeuroImage (2009) 45:758–68. 10.1016/j.neuroimage.2008.12.035 19166942

[33] Kimberley TJ Khandekar G Borich M. fMRI reliability in subjects with stroke. Exp Brain Res. (2008) 186:183–90. 10.1007/s00221-007-1221-8 18060395

[34] Kristo G Rutten GJ Raemaekers M de Gelder B Rombouts SA Ramsey NF. Task and task-free FMRI reproducibility comparison for motor network identification. Hum Brain Mapp. (2014) 35:340–52. 10.1002/hbm.22180 22987751 PMC6869575

[35] Gaxiola-Valdez I Goodyear BG. Origins of intersubject variability of blood oxygenation level dependent and arterial spin labeling fMRI: implications for quantification of brain activity. Magn Reson Imaging (2012) 30:1394–400. 10.1016/j.mri.2012.05.002 22795932

[36] Tjandra T Brooks JCW Figueiredo P Wise R Matthews PM Tracey I. Quantitative assessment of the reproducibility of functional activation measured with BOLD and MR perfusion imaging: implications for clinical trial design. NeuroImage (2005) 27:393–401. 10.1016/j.neuroimage.2005.04.021 15921936

[37] Bötzel K Tronnier V Gasser T. The differential diagnosis and treatment of tremor. Deutsch Ärztebl Int. (2014) 111:225–36. 10.3238/arztebl.2014.0225 24739887 PMC3991161

[38] WHO (2013). Atlas of MS 2013: Mapping Multiple Sclerosis Around the World. Available online at: http://www.msif.org/about-ms/publications-and-resources/

[39] Van der Walt A Buzzard K Sung S Spelman T Kolbe SC Marriott M. The occurrence of dystonia in upper-limb multiple sclerosis tremor. Mult Scler. (2015) 21, 1847–1855. 10.1177/1352458515577690 26014602

[40] Gallay MN Jeanmonod D Liu J Morel A. Human pallidothalamic and cerebellothalamic tracts: anatomical basis for functional stereotactic neurosurgery. Brain Struct Funct. (2008) 212:443–63. 10.1007/s00429-007-0170-0 18193279 PMC2494572

[41] Kobayashi K Katayama Y Kasai M Oshima H Fukaya C Yamamoto T. Localization of thalamic cells with tremor-frequency activity in Parkinson's disease and essential tremor. Acta Neurochir Suppl. (2003) 87, 137–139. 10.1007/978-3-7091-6081-7_29 14518541

[42] Magnin M Jeanmonod D Morel A Siegemund M Surgical control of the human thalamocortical dysrhythmia. Thalamus Related Syst. (2001) 1:81–9. 10.1016/S1472-9288(01)00002-4

[43] Obeso JA Rodriguez-Oroz MC Rodriguez M Lanciego JL Artieda J Gonzalo N. Pathophysiology of the basal ganglia in Parkinson's disease. Trends in Neurosciences (2000) 23:S8–19. 10.1016/S1471-1931(00)00028-8 11052215

[44] Tasker RR Lang AE Lozano AM. Pallidal and thalamic surgery for Parkinson's disease. Exp Neurol. (1997) 144:35–40. 10.1006/exnr.1996.6385 9126149

