# Supplementary material for: Novel Functional MRI Task for Studying the Neural Correlates of Upper Limb Tremor
Source: Front Neurol. 2018 Jul 2;9:513. doi: 10.3389/fneur.2018.00513 (PMC6036145; doi:10.3389/fneur.2018.00513)
Supplement: Supplementary file 1 [file Table_1.DOCX]

**SUPPLEMENTARY TABLE 1.** For each subject, the relative motion and number of slices removed due to excessive motion from the 400 slices dataset.

|  | | **Baseline** | | **6 weeks** | |
| --- | --- | --- | --- | --- | --- |
|  | | Relative motion | Removed slices | Relative motion | Removed slices |
| ***Subject 1*** | Median  Minimum  Maximum | 0.052 | 11 | 0.056 | 12 |
|  |  | 0.007 |  | 0.012 |  |
|  |  | 0.415 |  | 0.312 |  |
| ***Subject 2*** | Median  Minimum  Maximum | 0.090 | 4 | 0.086 | 6 |
|  |  | 0.006 |  | 0.013 |  |
|  |  | 0.332 |  | 0.511 |  |
| ***Subject 3*** | Median  Minimum  Maximum | 0.111 | 18 | 0.121 | 7 |
|  |  | 0.023 |  | 0.020 |  |
|  |  | 1.157 |  | 1.077 |  |
| ***Subject 4*** | Median  Minimum  Maximum | 0.154 | 21 | 0.128 | 16 |
|  |  | 0.014 |  | 0.013 |  |
|  |  | 1.285 |  | 0.875 |  |
| ***Subject 5*** | Median  Minimum  Maximum | 0.055 | 10 | 0.062 | 20 |
|  |  | 0.006 |  | 0.007 |  |
|  |  | 0.557 |  | 2.350 |  |
| ***Subject 6*** | Median  Minimum  Maximum | 0.070 | 18 | 0.053 | 9 |
|  |  | 0.012 |  | 0.001 |  |
|  |  | 0.231 |  | 0.181 |  |
| ***Subject 7*** | Median  Minimum  Maximum | 0.152 | 19 | 0.175 | 22 |
|  |  | 0.009 |  | 0.031 |  |
|  |  | 0.858 |  | 0.719 |  |
| ***Subject 8*** | Median  Minimum  Maximum | 0.077 | 19 | 0.088 | 18 |
|  |  | 0.012 |  | 0.015 |  |
|  |  | 0.532 |  | 0.393 |  |
| ***Subject 9*** | Median  Minimum  Maximum | 0.063 | 19 | 0.066 | 5 |
|  |  | 0.014 |  | 0.011 |  |
|  |  | 0.166 |  | 0.500 |  |
| ***Subject 10*** | Median  Minimum  Maximum | 0.107 | 20 | 0.124 | 28 |
|  |  | 0.014 |  | 0.011 |  |
|  |  | 0.741 |  | 0.810 |  |
| ***Subject 11*** | Median  Minimum  Maximum | 0.169 | 18 | 0.141 | 19 |
|  |  | 0.023 |  | 0.021 |  |
|  |  | 0.674 |  | 1.001 |  |
| ***Subject 12*** | Median  Minimum  Maximum | 0.160 | 30 | 0.121 | 23 |
|  |  | 0.033 |  | 0.020 |  |
|  |  | 1.194 |  | 0.609 |  |
